# Targeting TXNIP for neuroprotection: A novel approach to reducing inflammation and promoting recovery in ischemic stroke

**DOI:** 10.17305/bb.2024.11366

**Published:** 2024-12-13

**Authors:** Chongxin He, Yong Bao, Yong Xu, Jingjing Cheng, Xinxin Hu

**Affiliations:** 1 Department of Neurosurgery, Hefei Third People’s Hospital, Hefei Third Clinical College of Anhui Medical University, Hefei, Anhui, China; 2 Department of Neurology, Hefei Third People’s Hospital, Hefei Third Clinical College of Anhui Medical University, Hefei, Anhui, China; 3 Department of Science and Education, The Third People’s Hospital of Hefei, Hefei Third Clinical College of Anhui Medical University, Hefei, Anhui, China; 4 Anhui Province Key Laboratory of Occupational Health, Anhui Provincial People’s Hospital, Hefei, China

**Keywords:** Thioredoxin interacting protein, TXNIP, ischemic stroke, inflammation, angiogenesis, blood–brain barrier

## Abstract

Ischemic stroke often results in high mortality and significant disability. Current research primarily focuses on understanding neuroinflammation and cell death following a stroke to identify novel therapeutic targets. This study investigates the endothelial cell-specific role of Thioredoxin interacting protein (TXNIP) in ischemic stroke and its underlying molecular mechanisms both in vitro and in vivo. By targeting endothelial cells, we aim to determine how TXNIP knockdown promotes neuroprotection, enhances angiogenesis, and reduces inflammation post-stroke. In vitro, an oxygen-glucose deprivation (OGD) model using bEnd.3 cells simulated ischemic conditions. Cellular injury was evaluated through cell proliferation and angiogenesis assays, while dual immunofluorescence staining assessed ZO-1 and CD31 expression. Western blotting measured protein levels of TXNIP, nucleotide-binding oligomerization domain-like receptor protein 3 (NLRP3), ASC, pro-caspase-1, and interleukin-1β (IL-1β). In vivo, a middle cerebral artery occlusion (MCAO) mouse model was employed to mimic ischemic stroke. Brain injury was evaluated using triphenyltetrazolium chloride (TTC) and Nissl staining, and molecular changes in injury markers were assessed via Western blot analysis. In vitro, TXNIP knockdown promoted cell proliferation and angiogenesis, reduced inflammation, and decreased ZO-1 and CD31 fluorescence intensity. TXNIP knockdown also reversed OGD-induced upregulation of TXNIP, NLRP3, ASC, pro-caspase-1, and IL-1β. In vivo, TXNIP knockdown improved neurological recovery, reflected by lower Longa scores, increased Nissl body presence, and reduced infarct size. These findings suggest that TXNIP knockdown mitigates inflammation, enhances angiogenesis, and reduces cerebral damage following ischemic stroke. This provides valuable insights into potential endothelial cell-specific therapeutic strategies for stroke treatment.

## Introduction

Ischemic stroke, also known as cerebral infarction, is a clinical syndrome characterized by localized ischemic necrosis or softening of brain tissue caused by impaired blood circulation in the brain [1, 2]. Currently, the clinical treatment of ischemic stroke focuses on restoring blood flow to the cerebral arteries through intravenous thrombolysis and mechanical thrombectomy. These approaches aim to reduce ischemia in affected brain tissue and minimize subsequent hypoxic damage [3]. However, further research is essential to elucidate the underlying molecular mechanisms of ischemic stroke and to develop additional therapeutic strategies for mitigating brain tissue injury. In recent years, various signaling pathways have emerged as focal points in stroke research. The nucleotide-binding oligomerization domain-like receptor protein 3 (NLRP3) and the caspase-1-mediated pyroptosis pathway play pivotal roles in ischemic stroke-induced brain injury [4–6]. Furthermore, key factors in the classical inflammatory pathway, such as interleukin-1β (IL-1β) and interleukin-18 (IL-18), are critical for exploring the mechanisms that underlie treatment efficacy [7]. Thioredoxin-interacting protein (TXNIP) is a stress-response gene highly induced by early diabetes and hyperglycemia [8]. TXNIP was initially identified as an inhibitor of thioredoxin (TRX), impairing its ability to scavenge reactive oxygen species (ROS) [9, 10]. Recent studies have demonstrated that TXNIP release is associated with NLRP3 inflammasome activation and IL-1β production, particularly in contexts of diabetes and oxidative stress. Suppressing TXNIP expression has been shown to block the early pathology of diabetic retinopathy [11, 12]. Moreover, a recent study revealed that TXNIP deletion alleviates hepatic steatosis and inflammatory responses in a mouse model of non-alcoholic steatohepatitis (NASH) [13]. Based on these findings, we hypothesize that TXNIP, as an inflammatory protein, plays a critical role in regulating inflammation during the progression of inflammation-related diseases. It is well established that the inflammatory process following ischemic stroke onset is closely associated with disease progression and prognosis. However, the specific implications of targeting TXNIP in ischemic stroke remain poorly understood. In this study, we focus on the role of TXNIP in endothelial cells using bEnd.3 cells and animal models of ischemic stroke. By targeting TXNIP in endothelial cells, we aim to elucidate how its knockdown mitigates inflammation, enhances angiogenesis, and protects against brain injury. Our findings may offer novel insights and therapeutic targets for the clinical treatment of ischemic stroke.

## Materials and methods

### Cell culture and transfection

Murine brain microvascular endothelial cells (bEnd.3) were obtained from Procell Life Science & Technology Co., Ltd. (Wuhan, China). These cells were cultured in a 1:1 mixture of DMEM and Ham′s F12 medium, supplemented with 10% fetal bovine serum (FBS) and penicillin/streptomycin.TXNIP negative control siRNA (siNC) and specific siRNAs were developed by Shanghai Shengbo Biomedical Co., Ltd. (China). We designed three TXNIP-targeting siRNA sequences, which included siTXNIP-1 (sense: 5′-CGAUGUGGACGACUCUCA AGA-3′; antisense: 5′-UUGAGAGUCGUCCACAUCGUC-3′), siTXNIP-2 (sense: 5′-GGAUCUAGUGGAUGUCAAUAC-3′; antisense: 5′-AUUGACAUCCACUAGAUCCAU-3′), and siTXNIP-3 (sense: 5′-AGACCAAAGUGUUCACUCAGA-3′; antisense: 5′-UGAGUGAACACUUUGGUCUGG-3′). Transient transfections were carried out using Lipofectamine 3000 (Thermo Fisher Scientific, USA) in accordance with the manufacturer′s protocol. Western blotting was performed to evaluate transfection efficiency. Additionally, lentiviral vectors and packaging plasmids (Beyotime, Shanghai, China) were co-transfected into HEK293 cells.

### Cell grouping

To induce oxygen-glucose deprivation (OGD), the cell culture medium was replaced with glucose-free DMEM (Gibco, USA). The cells were then placed in a modular incubator chamber set to 1% O_2_, 5% CO_2_, and 94% N_2_ for 30 min at room temperature. After 12 h of OGD, the cells were transferred to glucose-containing DMEM (without FBS) and incubated under normoxic conditions for an additional 12 h to allow reoxygenation. Based on the treatments, the cells were divided into four experimental groups: Blank (no treatment), OGD, OGD + siNC, and OGD + siTXNIP.

### Cell counting kit-8 (CCK8) assay

Cells were seeded in 96-well plates at a density of 5×10^3^ cells per well and incubated for 24 h at 37 ^∘^C in a 5% CO_2_ atmosphere. After the incubation period, 10 µL of CCK-8 reagent (Dojindo, Japan) was added to each well, and the plates were incubated for an additional 2 h. Absorbance at 450 nm was then measured using a microplate reader (Bio-Rad, Berkeley, CA, USA).

### Tube formation assay

To evaluate the angiogenic potential, bEnd.3 cells and HUVECs were co-cultured. Matrigel (BD Biosciences) was thawed overnight at 4 ^∘^C, and 50 µL was dispensed into each well of a 96-well plate. The plate was incubated at 37 ^∘^C for 30 min to allow the Matrigel to solidify, followed by an additional 60 min incubation at 37 ^∘^C. HUVECs (3×10^5^ cells/well) were then seeded and cultured with supernatants from bEnd.3 cells from each experimental group for 4 h. HUVEC angiogenesis was subsequently assessed and documented using light microscopy.

### Enzyme-linked immunosorbent assay (ELISA)

The concentrations of vascular endothelial growth factor (VEGFA), tumor necrosis factor α (TNF-α), and interleukin 6 (IL-6) were quantified using ELISA kits, following the manufacturer’s protocols. The VEGFA, TNF-α, and IL-6 assay kits were obtained from by Abscience (Shanghai, China).

### Western blot assay

bEnd.3 endothelial cells or mouse brain tissues were lysed in RIPA lysis buffer containing protease and phosphatase inhibitors (Beyotime, China). Protein concentrations were determined using a BCA kit (Abcam, Cambridge, UK). Equal amounts of protein samples were separated by 10% SDS-PAGE, transferred onto 0.22-µm PVDF membranes (Millipore, USA), and blocked with 5% nonfat dried milk. The membranes were incubated overnight at 4 ^∘^C with the following primary antibodies: TXNIP (ab318848, Abcam, Cambridge, UK), TRX1 (ab283400, Abcam, Cambridge, UK), NLRP3 (ab263899, Abcam, Cambridge, UK), ASC (ab283684, Abcam, Cambridge, UK), pro-caspase-1 (ab179515, Abcam, Cambridge, UK), and IL-1β (ab315804, Abcam, Cambridge, UK). The next day, membranes were incubated with appropriate secondary antibodies (Santa Cruz, CA, USA). Protein bands were visualized using enhanced chemiluminescence (ECL) reagents (Beyotime, Shanghai, China) and detected with the Tanon 5200 Multi imager (Tanon Science & Technology, Shanghai, China). Grayscale values of the bands were analyzed using ImageJ software.

### Animal experiments

Twenty-four male C57BL/6 mice (22–27 g, 8–10 weeks old) (License Number: SCXK 20220006, Shandong Experimental Animal Center, China) were housed under specific pathogen-free (SPF) conditions with ad libitum access to food and water. After a 7-day acclimatization period, the mice were randomly assigned to one of four groups: Sham group, Model group, Model + shNC group, and Model + shTXNIP group. All animal care procedures and experimental protocols strictly adhered to the guidelines of the China Council on Animal Care and Use and received approval from the Ethics Committee of Anhui Medical University. An ischemic stroke model was established in mice using the middle cerebral artery occlusion (MCAO) technique. Briefly, mice were anesthetized with 5% isoflurane for induction and maintained under 2% isoflurane. A midline cervical incision (approximately 2 cm in length) was made to expose the right common carotid artery. Using a stereomicroscope, the fascia surrounding the blood vessels at the bifurcation of the internal and external carotid arteries was carefully excised, and the external carotid artery was isolated. A suture was then advanced along the external carotid artery into the internal carotid artery until slight resistance was detected, indicating successful occlusion. Ischemia was maintained for 1 h, after which the suture was retracted to restore blood flow, and the incision was sutured. Twenty-four hours after MCAO induction, the mice were euthanized, and brain tissues were harvested for analysis. Notably, one week prior to modeling, lentivirus carrying the target gene or NC was injected into the tail vein of the mice.

### Neurological behavior assessment

The Longa scoring method (Longa et al., 1989) is a commonly used behavioral assessment method for animal models of hypoxic-ischemic diseases. The criteria are as follows: normal activity without neurological symptoms (0 points); paralyzed front paws cannot be fully extended (1 point); turn in circles toward the paralyzed side while walking (2 points); (4) tilt toward the paralyzed side while walking (3 points); (5) unable to walk automatically and experiencing loss of consciousness (4 points). Higher scores indicate more severe neurological deficits.

### 2,3,5-Triphenyltetrazolium chloride (TTC) staining

The brain tissue was initially frozen at –80 ^∘^C for approximately 5 min and subsequently sectioned into 2-mm slices. These slices were stained with 2% TTC (Solarbio, Beijing, China) and incubated in a water bath at 37 ^∘^C for 30 min. Following staining, the sections were fixed in 4% paraformaldehyde for 24 h. Images were then captured to identify the infarcted regions, where white areas corresponded to infarcted brain tissue and red areas indicated normal tissue.

### Nissl staining

The brains were post-fixed with 4% paraformaldehyde at room temperature overnight, dehydrated through a graded alcohol series, and embedded in paraffin. Sections of 4-µm thickness were prepared and stained with Nissl stain (Beyotime, Shanghai, China) using toluidine blue. The stained sections were examined under an Olympus BX51 microscope (Olympus, Center Valley, PA, USA) at ×200 magnification.

### Dual immunofluorescence staining

The tissues were fixed, dehydrated, and sectioned into 4-µm slices. Cell climbing slices were prepared for standard immunofluorescence. Briefly, the sections or cell climbing slices were treated with 4% paraformaldehyde and 0.5% Triton X-100 for 10 min each at room temperature. Subsequently, the cells were incubated with primary antibodies: Alexa Fluor^®^ 594 anti-CD31 (1:50, Abcam, UK) and ZO-1 labeled with FITC (1:200, ThermoFisher, USA). For tissue sections, the primary antibodies used were ZO-1 labeled with Alexa Fluor^®^ 594 and CD31 labeled with FITC. Goat anti-rabbit IgG conjugated with horseradish peroxidase (antibody-online, Germany) was then applied to the specimens and incubated at 37 ^∘^C for 1 h. Finally, the sections were counterstained with DAPI (Invitrogen, Germany), and images were acquired using an Olympus microscope (Tokyo, Japan).

### Ethical statement

All experiments performed in this study adhered to the ethical guidelines of the Declaration of Helsinki and was approved by the Ethics Committees of Hefei Third Clinical College of Anhui Medical University (No. 2020LLW012).

### Statistical analysis

Statistical analysis was performed using SPSS 20.0 (IBM, New York, USA). Group comparisons were conducted using one-way analysis of variance (ANOVA), followed by Duncan’s test for multiple comparisons. Data are presented as mean ± SD, with statistical significance set at *P* < 0.05. All trials were conducted in triplicate.

## Results

### The effects of TXNIP on proliferation and angiogenesis in bEnd.3 cells after OGD

**Figure 1.**
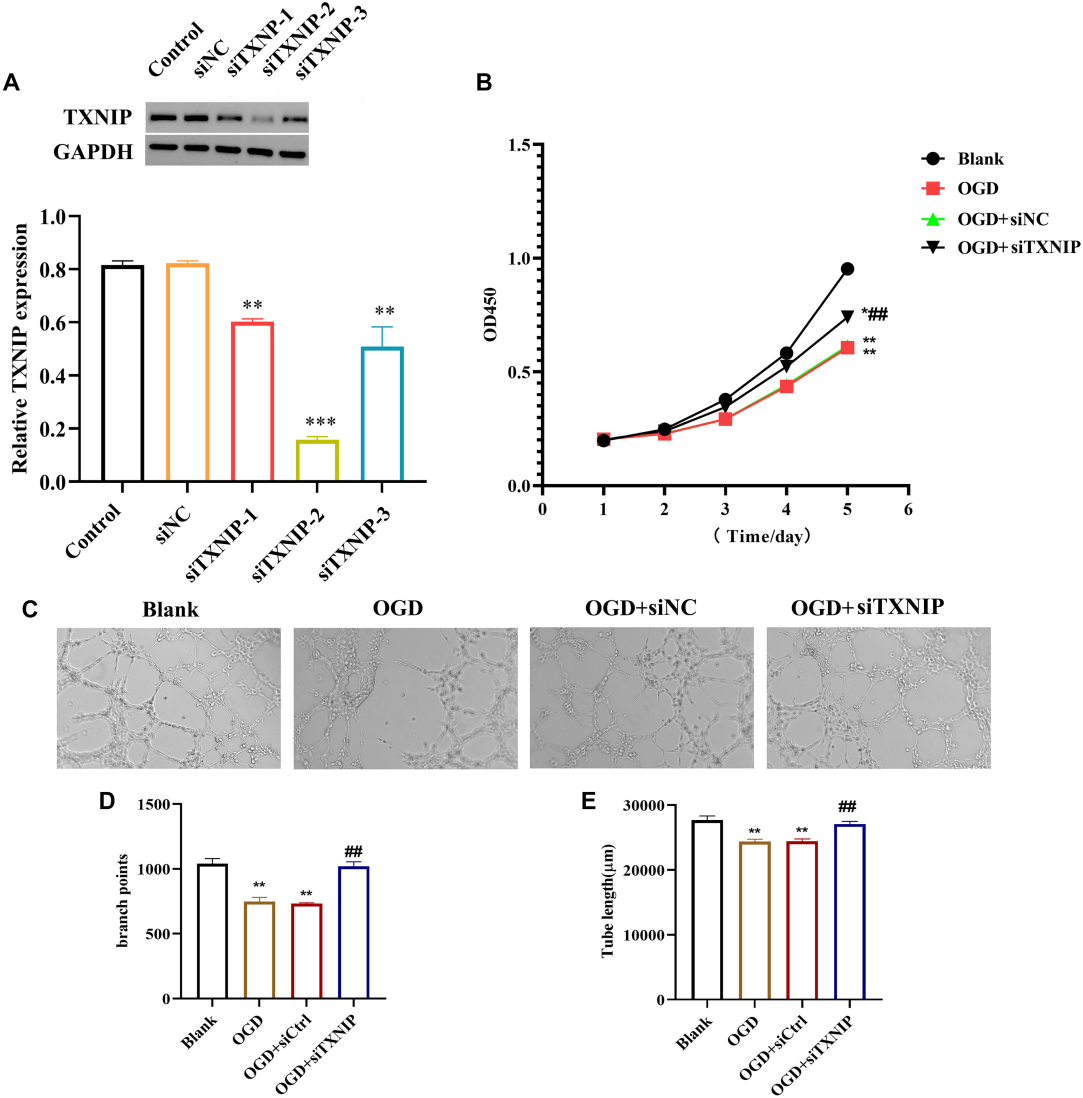
**TXNIP knockdown enhanced cells proliferation and vascularization capacity.** (A) The expression of TXNIP was suppressed after different siRNA transfection. vs Control or siNC group, ^**^P < 0.01, ^***^P < 0.001; (B) CCK8 result revealed the cells proliferation was enhanced in the OGD+siTXNIP group; (C) Tube formation assay presented the enhanced vascularization capacity after transfecting with siTXNIP; (D) Bar graph presented the branch points; (E) Bar graph presented the tube length. vs Blank group, *P < 0.05, **P < 0.01; vs OGD or OGD+siNC group, ^##^*P* < 0.01. TXNIP: Thioredoxin interacting protein; OGD: Oxygen-glucose deprivation; CCK8: Cell counting kit-8.

**Figure 2.**
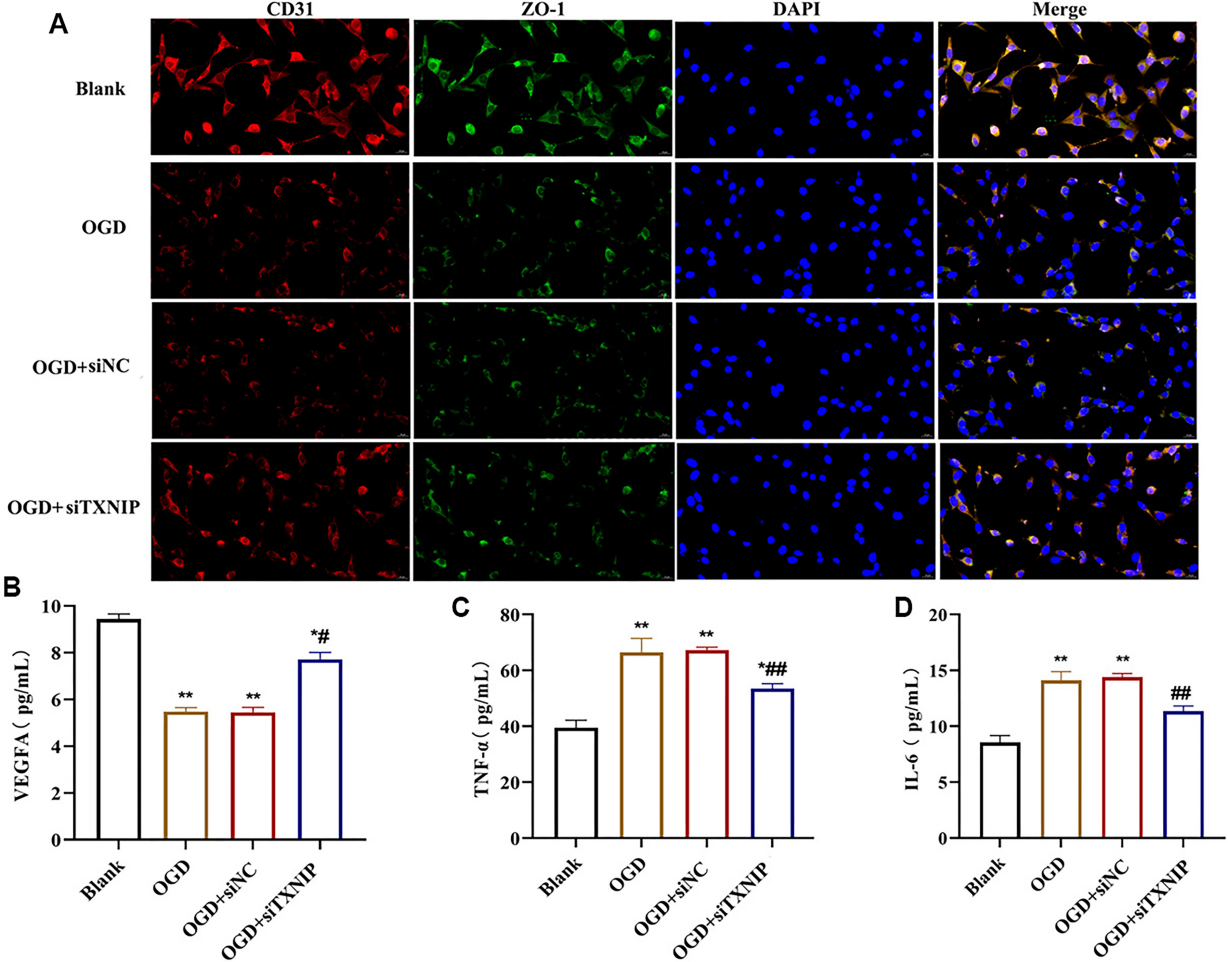
**The effects of TXNIP on angiogenesis and inflammation in bEnd.3 cells.** (A) The expression of CD31 and ZO-1 were detected by double IF staining; (B) VEGFA content in cells was detected by ELISA, and presented significant increased after TXNIP knockdown; (C) TNF-α and IL-1β was detected by ELISA, and presented significant decreased after TXNIP knockdown. vs Blank group, *P < 0.05, **P < 0.01; vs OGD or OGD+siNC group, ^#^*P* < 0.05, ^##^*P* < 0.01. TXNIP: Thioredoxin interacting protein; OGD: Oxygen-glucose deprivation; IL-1β: Interleukin-1β; ELISA: Enzyme-linked immunosorbent assay; VEGFA: Vascular endothelial growth factor; TNF-α: Tumor necrosis factor α.

We first evaluated the transfection efficiency of TXNIP in bEnd.3 endothelial cells. As shown in Figure 1A, the expression level of TXNIP in the siNC group was comparable to that in the Control group, indicating that siNC had no effect on TXNIP expression. In contrast, different siRNAs showed varying knockdown efficiencies, with siTXNIP-2 inducing the most significant reduction in TXNIP expression (*P* < 0.01). Therefore, siTXNIP-2 was selected as the target for RNA interference. Next, we assessed cell proliferation and angiogenesis in bEnd.3 cells following OGD treatment. Cell proliferation in the OGD and OGD+siNC groups was significantly reduced compared to the Blank group. However, the OGD+siTXNIP group exhibited a markedly higher OD value compared to the OGD and OGD+siNC groups (*P* < 0.05, Figure 1B). Furthermore, the OGD+siTXNIP group displayed an increased number of vessel segments compared to the OGD and OGD+siNC groups (Figure 1C–1E). Dual immunofluorescence staining revealed that CD31 and ZO-1 were expressed in the cytoplasm. The fluorescence intensity of CD31 and ZO-1 decreased in the OGD and OGD+siNC groups but was enhanced in the OGD+siTXNIP group (Figure 2A). Similarly, the VEGFA levels were reduced in the OGD and OGD+siNC groups but significantly elevated in the OGD+siTXNIP group (*P* < 0.05, Figure 2B). These findings demonstrate that TXNIP knockdown exerts a protective effect on bEnd.3 cell proliferation and angiogenic capacity under OGD conditions.

### TXNIP knockdown improved inflammation in bEnd.3 with OGD

**Figure 3.**
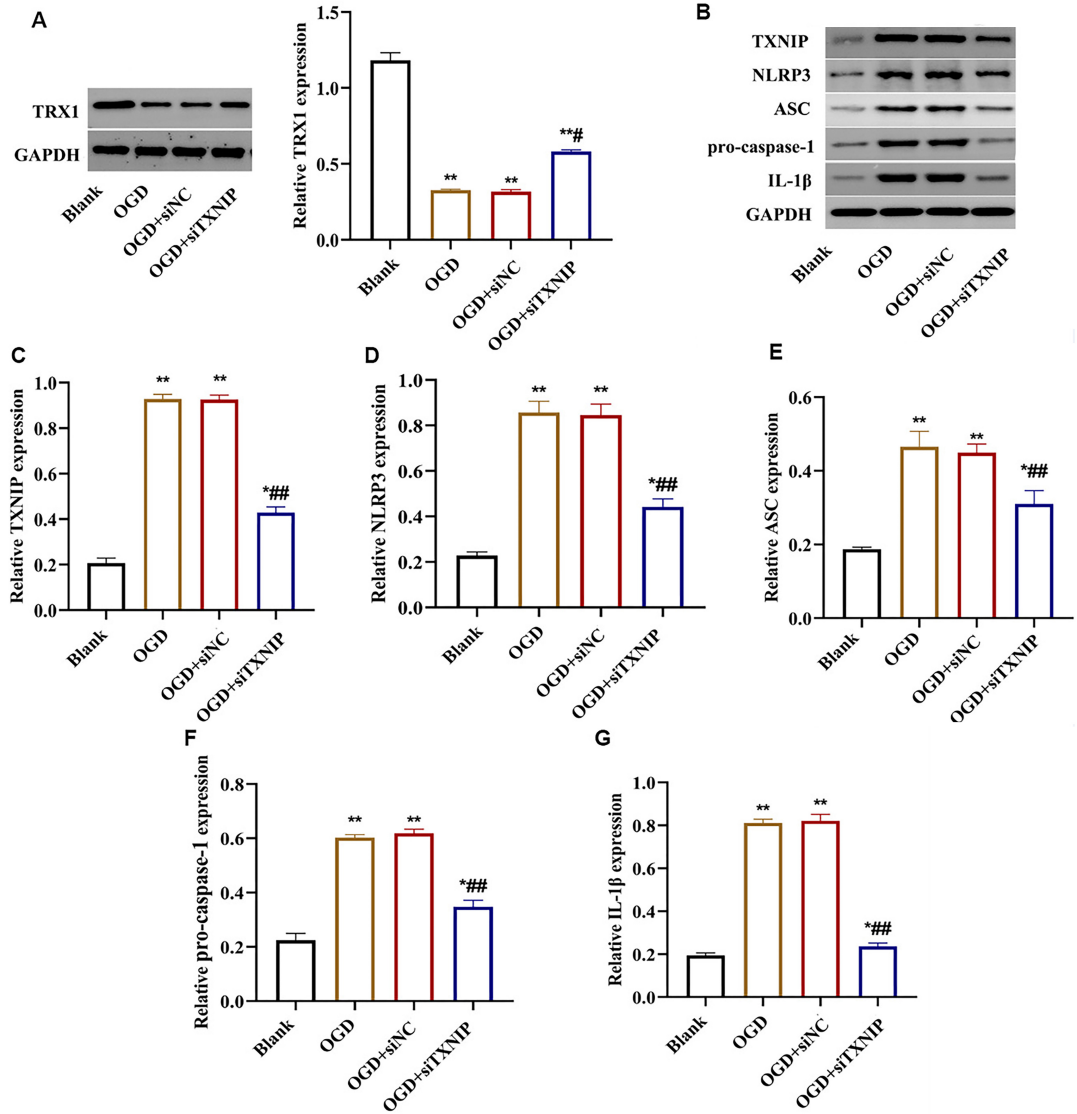
**TXNIP knockdown suppressed the activation of NLRP3 inflammasome in bEnd.3 cells.** (A) TRX1 protein expression level was increased after TXNIP knockdown; (B) Western blot results presented the changes of TXNIP, NLRP3, ASC, pro-caspase-1, and IL-1β proteins levels after TXNIP knockdown. Bar graph revealed the decreased express of TXNIP (C), NLRP3 (D), ASC (E), pro-caspase-1 (F), and IL-1β (G) proteins. vs Blank group, *P < 0.01, **P < 0.01, vs OGD or OGD+ siNC group, ^#^*P* < 0.01, ^##^*P* < 0.01. TXNIP: Thioredoxin interacting protein; OGD: Oxygen-glucose deprivation; NLRP3: Nucleotide-binding oligomerization domain-like receptor protein 3; 1β: Interleukin-1β.

Inflammation is a critical component of ischemic stroke. ELISA results showed that TNF-α and IL-6 levels were significantly elevated in the OGD and OGD+siNC groups compared to the control group. Notably, the TNF-α and IL-6 levels in the OGD+siTXNIP group were significantly reduced compared to the OGD and OGD+siNC groups (*P* < 0.05, Figure 2C and 2D). Western blot results demonstrated that TRX1 expression levels decreased in the OGD and OGD+siNC groups compared to the Blank group but increased significantly in the OGD+siTXNIP group (*P* < 0.05, Figure 3A). Importantly, the expression levels of NLRP3, ASC, pro-caspase-1, and IL-1β proteins were elevated in both the OGD and OGD+siNC groups. However, these protein levels were significantly reduced in the OGD+siTXNIP group compared to the OGD and OGD+siNC groups, though they remained higher than those in the Blank group (*P* < 0.05, Figure 3B–3G).

### TXNIP knockdown alleviates brain injury in mice with ischemic stroke

**Figure 4.**
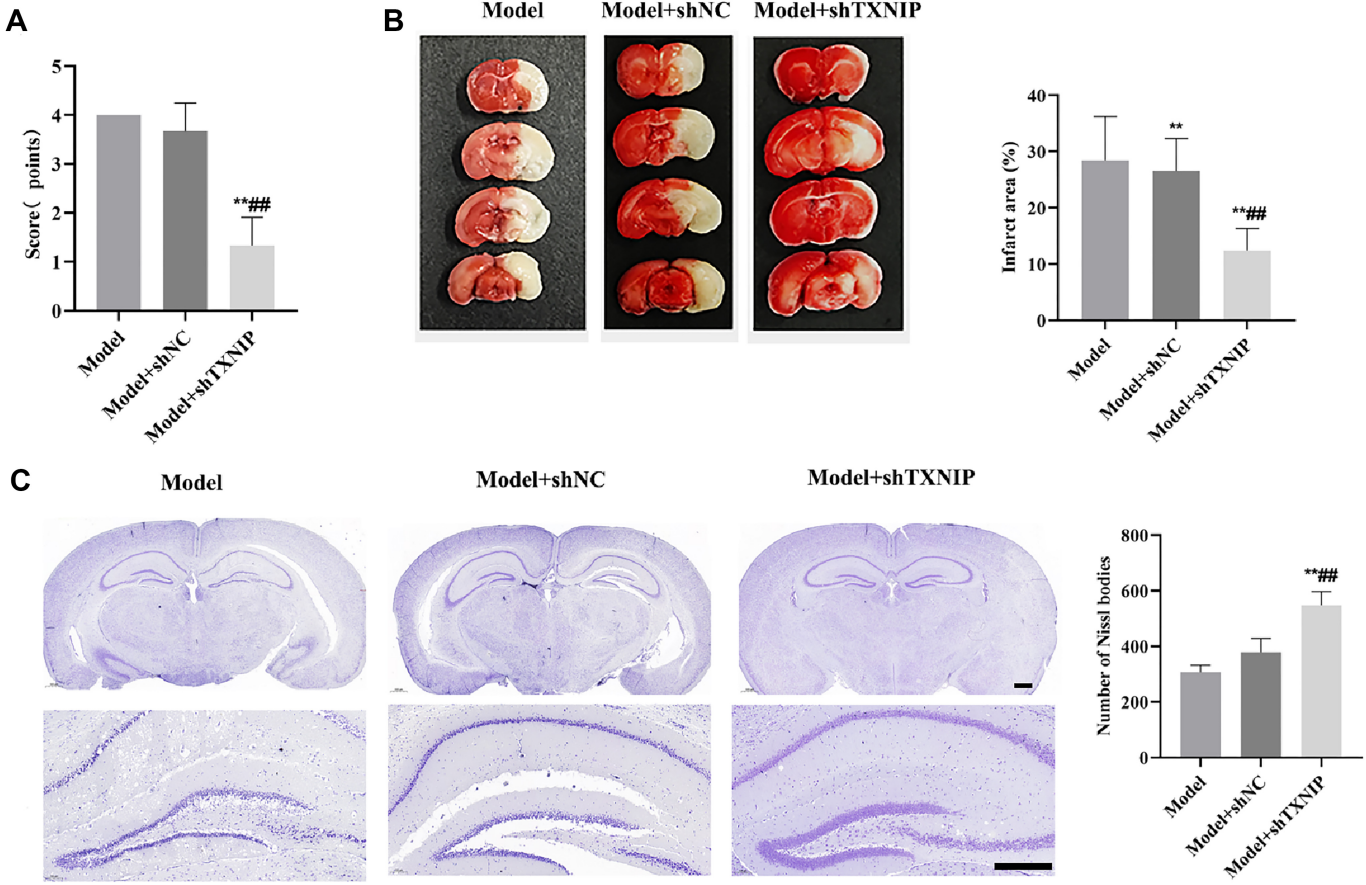
**TXNIP knockdown alleviates brain injury in ischemic stroke mice model.** (A) The neurobehavioral scores were assessed by Longa method; (B) The volume of cerebral infarction was detected by TTC staining, and the Model+shTXNIP presented obviously improving; (C) Nissl stain was used to observe the changes of nissl body in the brain tissue. vs Model group, **P < 0.01; vs Model+shNC group, ^##^*P* < 0.01. Scale bars: 500 µm. TXNIP: Thioredoxin interacting protein; TTC: Triphenyltetrazolium chloride.

To further verify the effect of TXNIP in ischemic stroke in vivo, we established a MACO model in mice. As shown in Figure 4A, there were no significant differences in neurobehavioral scores between the Model group and the Model + shNC group. However, the Model + shTXNIP group exhibited a significant reduction in neurobehavioral scores compared to the Model and Model + shNC groups (*P* < 0.01). TTC staining results (Figure 4B) showed no significant differences in the infarct area of mouse brain tissue between the Model and Model + shNC groups. In contrast, the infarct area was significantly reduced in the Model + shTXNIP group compared to both the Model and Model + shNC groups. Nissl staining results (Figure 4C) revealed that the number of Nissl bodies in brain tissue was decreased in the Model and Model + shNC groups. Conversely, the Model + shTXNIP group demonstrated an increased number of Nissl bodies compared to the Model and Model + shNC groups.

### TXNIP knockdown promoted the revascularization of blood vessels in mice with ischemic stroke

**Figure 5.**
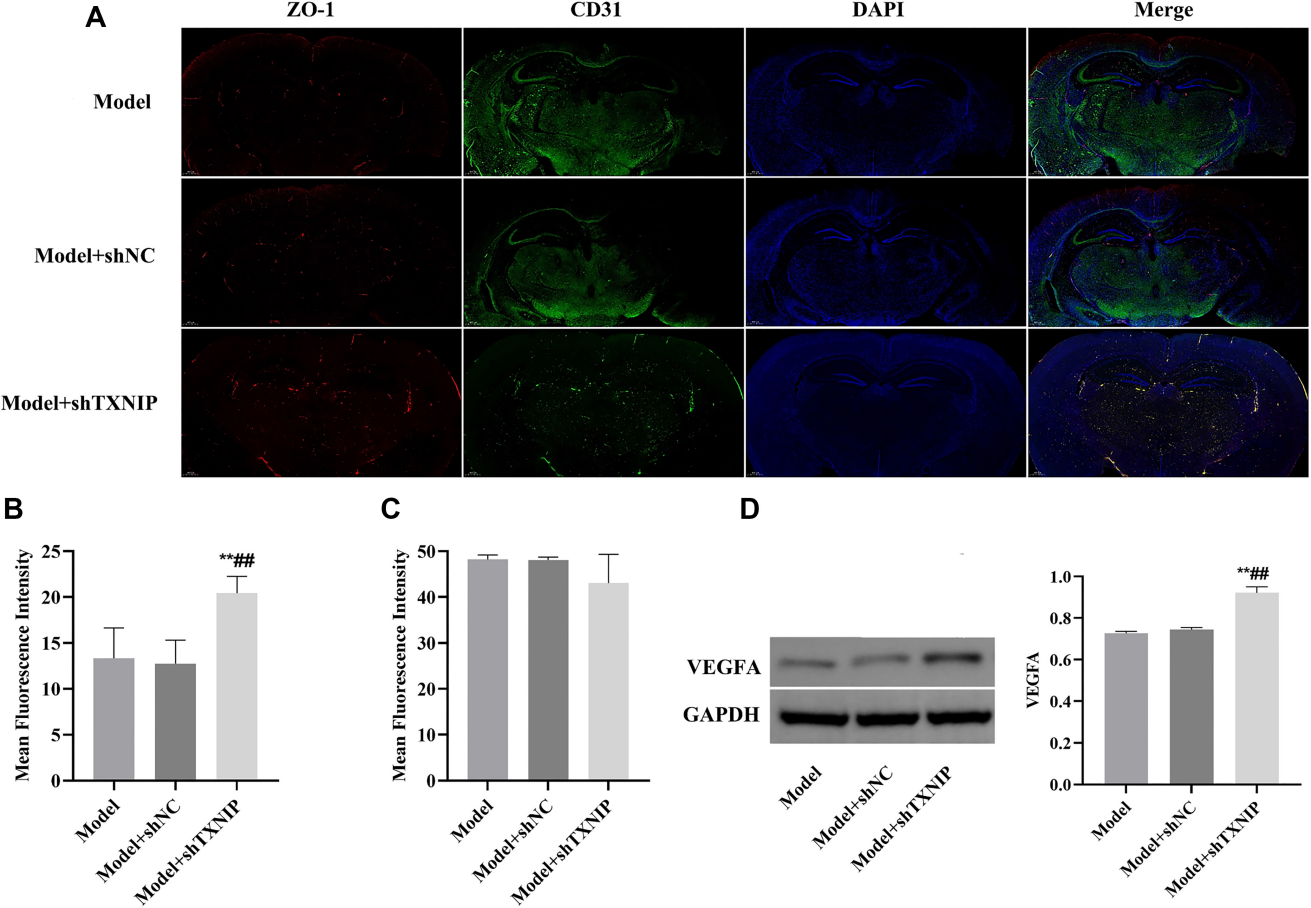
**TXNIP knockdown promoted the revascularization of blood vessels.** (A) Dual immunofluorescence detection of CD31 and ZO-1 expression in the brain tissue; (B) Bar graph revealed the mean fluorescence intensity of ZO-1; (C) Bar graph revealed the mean fluorescence intensity of CD-31; (D) The expression level of VEGFA proteins was detected by western bolt. vs Model group, **P < 0.01; vs Model+shNC group, ^##^*P* < 0.01. TXNIP: Thioredoxin interacting protein; VEGFA: Vascular endothelial growth factor.

Immunofluorescence results indicated that the fluorescence intensity of CD31 in the brain tissue of the Model + shTXNIP group was higher compared to the Model and Model+shNC groups, whereas the fluorescence intensity of ZO-1 showed no significant increase (Figure 5A–5C). Western blot analysis revealed a significant increase in VEGFA protein levels in the Model + shTXNIP group compared to the Model and Model+shNC groups (*P* < 0.05, Figure 5D).

### TXNIP knockdown alleviates brain injury in mice with ischemic stroke by inhibiting the activation of NLRP3 inflammasome

**Figure 6.**
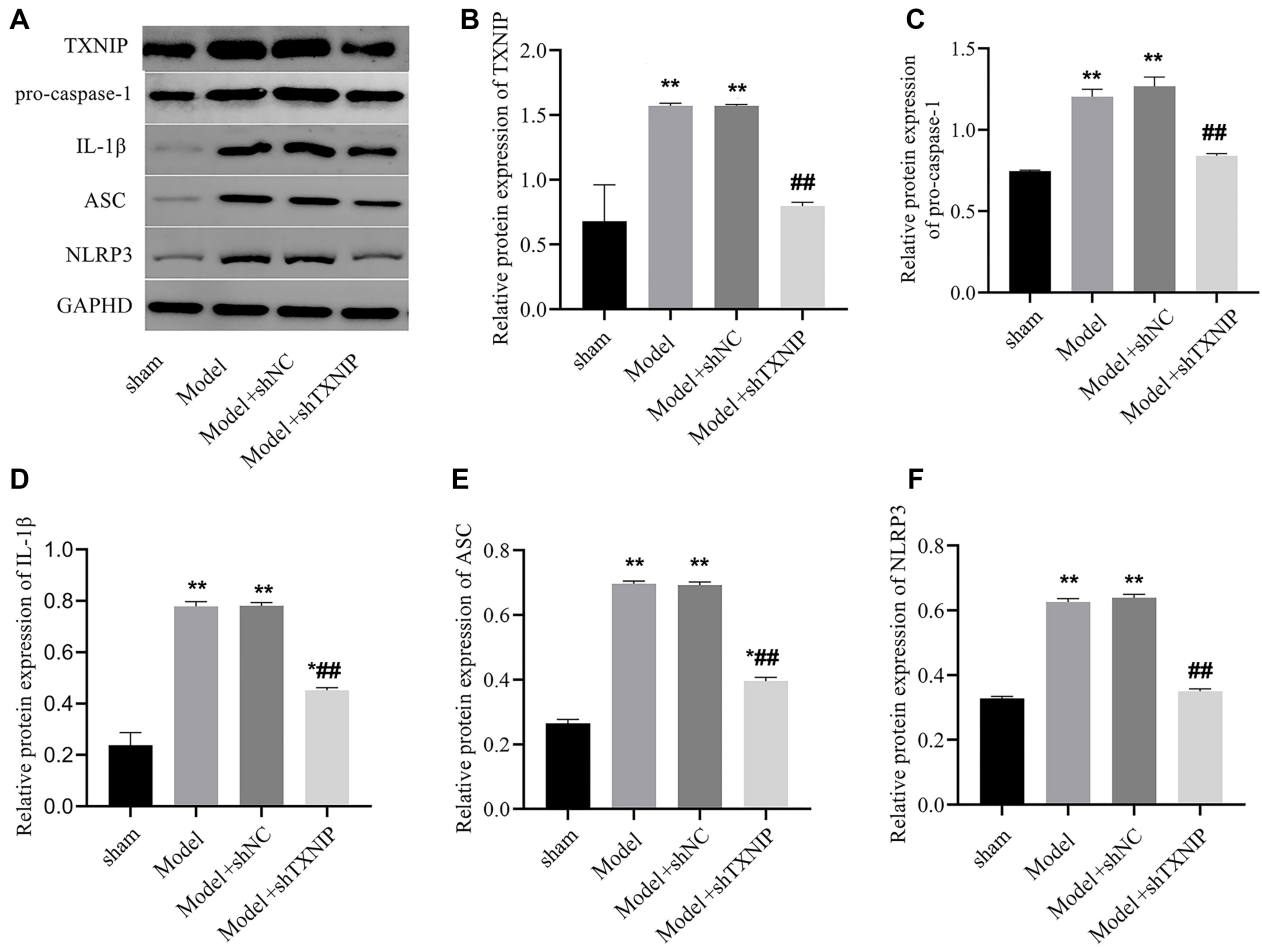
**TXNIP knockdown suppressed the expression of NLPR3.** (A) The expression levels of TXNIP, pro-caspase-1, IL-1β, ASC, and NLRP3 proteins were detected by western bolt. Bar graphs revealed the expression levels of TXNIP (B), pro-caspase-1 (C), IL-1β (D), ASC (E), and NLRP3 (F) proteins. vs sham group, *P < 0.01, **P < 0.01, vs Model or Model + shNC group, ^##^*P* < 0.01. TXNIP: Thioredoxin interacting protein; NLRP3: Nucleotide-binding oligomerization domain-like receptor protein 3; 1β: Interleukin-1β.

Next, we performed Western blot analysis on mouse brain tissue. Compared to the sham group, expression levels of key inflammasome-related proteins—including TXNIP, NLRP3, ASC, pro-caspase-1, and IL-1β—were significantly upregulated in both the Model and Model+shNC groups, suggesting that activation of the NLRP3 inflammasome pathway is closely associated with ischemic brain injury. However, TXNIP knockdown in the Model+shTXNIP group led to a marked reduction in these proteins compared to the Model and Model+shNC groups. This indicates that TXNIP plays a critical role in promoting NLRP3 inflammasome activation during ischemic stroke. The significant reduction in NLRP3, ASC, pro-caspase-1, and IL-1β levels in the Model+shTXNIP group (*P* < 0.05, Figure 6A–6F) highlights the protective effect of TXNIP knockdown in alleviating neuroinflammation and underscores its potential as a therapeutic target for mitigating ischemic stroke-induced brain injury. These findings emphasize the importance of targeting TXNIP to inhibit NLRP3 inflammasome activation and reduce inflammatory damage in ischemic conditions.

## Discussion

A previous study revealed that TXNIP aggravates oxidative stress injury by regulating the MAPK-Nrf2 axis in ischemic stroke [14]. However, there is still no clear evidence confirming the correlation between TXNIP and the NLRP3 inflammasome in ischemic stroke. Recent studies have demonstrated that inhibiting the NLRP3 inflammasome and enhancing autophagy levels can markedly mitigate stroke-induced damage [15–17]. Consequently, contemporary research on protective mechanisms against stroke injury has increasingly focused on regulating the NLRP3 inflammasome. Additionally, recent findings have shown that TXNIP is linked to the activation of the NLRP3 inflammasome in various diseases, promoting inflammatory processes [18–20]. In the present study, we found that TXNIP knockdown enhanced cell proliferation, promoted angiogenesis, suppressed cellular inflammation, and attenuated brain damage in ischemic stroke mouse models. To identify the most effective siRNA for TXNIP knockdown, we designed three siRNAs and transfected bEnd.3 cells with OGD. Among the siRNAs tested, siRNA-2 was found to be the most effective and was therefore used in subsequent experiments. We first investigated the changes in cell proliferation and angiogenesis following TXNIP knockdown. The results revealed that TXNIP knockdown had a significant promoting effect on these processes. VEGF, particularly VEGFA, plays a pivotal role in regulating angiogenesis [21]. VEGF facilitates vasodilation, enhances vascular permeability, stimulates endothelial cell proliferation and migration, accelerates neovascularization, improves cerebral blood flow, and mitigates brain injury [22, 23]. In our study, the increased expression of VEGFA further demonstrated the pro-angiogenic effects of TXNIP knockdown. These findings align with previous studies highlighting the role of TXNIP in angiogenesis. For instance, Shen et al. [24] reported that TXNIP knockdown increased angiogenesis in diabetic mice. Similarly, Song et al. [25] demonstrated that SIRT6 inhibited microglia activation and promoted angiogenesis in cerebral ischemia by suppressing TXNIP. Collectively, these studies underscore the critical role of TXNIP in angiogenesis and its potential as a therapeutic target in ischemic stroke.

CD31 serves as a critical biomarker for angiogenesis [26]. ZO-1, a tight junction protein, is predominantly located at the tight junctions between epithelial and endothelial cells and is essential for maintaining BBB integrity [27]. CD31 also plays a role in stabilizing BBB integrity [28]. In our study, data from the OGD model showed that TXNIP knockdown upregulated the expression levels of both CD31 and ZO-1. In in vivo experiments, TXNIP knockdown increased the presence of Nissl bodies and attenuated the infarct area. Additionally, the fluorescence intensity of CD31 in brain tissue was enhanced. However, ZO-1 fluorescence intensity showed no significant enhancement. We hypothesize that this discrepancy may arise from multiple factors. Previous studies have indicated that excessive production of inflammatory cytokines and overactivation of glia can lead to ZO-1 degradation [29]. Moving forward, we aim to investigate the specific effects of TXNIP knockdown on ZO-1 expression. The inflammasome is a multiprotein complex consisting of cytoplasmic receptor proteins, the adaptor protein ASC, and downstream pro-caspase-1 [30]. Numerous studies have demonstrated the involvement of NLRP3 inflammasomes in both neuronal cells and microglia, with significant implications for stroke pathology [31, 32]. Following a stroke, oxidative stress, mitochondrial damage, and disrupted ion homeostasis activate Toll-like receptors (TLRs) and NOD-like receptors (NLRs) [33]. This activation facilitates the assembly of the NLRP3 inflammasome complex, which includes the NLRP3 protein, ASC (apoptosis-associated speck-like protein containing a CARD), and pro-caspase-1 [34]. Activated NLRP3 inflammasomes subsequently trigger Caspase-1, catalyzing the maturation of pro-inflammatory cytokines pro-IL-1β and pro-IL-18. The secretion of IL-1β and IL-18 significantly exacerbates the inflammatory response [31]. In our study, we assessed the expression of NLRP3, ASC, pro-caspase-1, and IL-1β proteins both in vivo and in vitro. Our findings showed that these protein levels were elevated in the OGD or model group, with siNC treatment having no effect. As expected, TXNIP knockdown reduced the levels of NLRP3, ASC, pro-caspase-1, and IL-1β proteins, indicating that TXNIP’s role in ischemic stroke is associated with the NLRP3 inflammasome pathway. However, this study has several limitations. First, while we investigated the effects of TXNIP knockdown, we did not include treatment with an NLRP3 inhibitor. Additionally, although TXNIP is significantly upregulated in neurons during ischemic conditions and contributes to neurodegeneration, our study did not include experiments specifically targeting TXNIP knockdown in neuronal cells. Second, while we measured inflammatory factor levels in vitro, we did not assess their levels in vivo. Finally, the relationship between TXNIP knockdown and ZO-1 expression requires further investigation through additional in vivo experiments.

## Conclusion

In conclusion, TXNIP knockdown can suppress inflammation, promote cell proliferation, and enhance angiogenesis in vitro, thereby mitigating cerebral damage in ischemic stroke mouse models. This specific effect appears to be associated with the regulation of NLRP3 inflammasomes. Overall, this study offers a novel insight into potential treatment strategies for ischemic stroke.

## Data Availability

All datasets used and/or analyzed during the study are available from the corresponding author on reasonable request.
